# Visceral adipose tissue and osteoarthritis, a two-sample Mendelian randomized study

**DOI:** 10.3389/fmed.2023.1324449

**Published:** 2024-01-05

**Authors:** Yinzhen Zhang, Yanpeng Wang, Ji Xu, Zhengyan Wang, Wenhai Zhao, Changwei Zhao

**Affiliations:** ^1^Department of Traditional Chinese Medicine, Changchun University of Chinese Medicine, Changchun, China; ^2^Department of Spinal Orthopedics, Weifang Hospital of Traditional Chinese Medicine, Weifang, China; ^3^Department of Orthopedics, Affiliated Hospital of Changchun University of Chinese Medicine, Changchun, China

**Keywords:** obesity, visceral adipose tissue, osteoarthritis, Mendelian randomization, two-sample MR

## Abstract

**Background:**

The relationship between visceral adipose tissue and osteoarthritis is not clear. The purpose of our study was to explore the relationship between visceral adipose tissue and osteoarthritis.

**Methods:**

We used a two-sample Mendelian randomization method to select single-nucleotide polymorphisms (SNPs) significantly associated with visceral adipose tissue as instrumental variables to explore the relationship between visceral adipose tissue and all osteoarthritis, hand osteoarthritis, hip osteoarthritis, knee osteoarthritis, and spine osteoarthritis. The reliability of the results was tested using sensitivity analysis.

**Results:**

Our findings indicated that visceral adipose tissue was associated with all osteoarthritis, hip osteoarthritis, knee osteoarthritis, and spine osteoarthritis (all osteoarthritis: OR = 1.399, 95% CI: 1.335–1.467, *p* = 7.95e-44; hip osteoarthritis: OR = 1.399, 95% CI: 1.284–1.524, *p* = 1.41e-14; knee osteoarthritis: OR = 1.794, 95% CI: 1.662–1.937, *p* = 1.33e-50; and spine osteoarthritis: OR = 1.445, 95% CI: 1.314–1.589, *p* = 2.89e-14). Sensitivity analysis demonstrated the reliability of these results.

**Conclusion:**

Our study suggests that genetically predicted visceral adipose tissue is associated with osteoarthritis. Reducing the accumulation of visceral adipose tissue could potentially have an impact on the incidence of osteoarthritis.

## 1 Introduction

Osteoarthritis (OA) is a chronic degenerative disease characterized by cartilage degeneration, subchondral bone changes, and synovitis, primarily affecting the hip, knee, hand, and other joints (1). It has a high global prevalence (2) and ranks fifth among all causes of disability worldwide, posing a significant threat to human health (3). Treating OA includes early pain management and end-stage joint replacement, but the high cost of treatment imposes a significant burden on society and individuals (4). Although the mechanism and risk factors of OA are not fully understood, it is generally believed to be closely related to age, obesity, and other factors (5).

Obesity has been shown to be closely associated with the pathogenesis of various diseases (6–8). In these studies, obesity is often assessed using human indicators such as BMI, waist-to-hip ratio, waist circumference, and hip circumference. However, due to the heterogeneity of obesity, there are considerable individual differences in body fat distribution and metabolic characteristics, even among individuals with the same body mass index (BMI) (9). Thus, BMI or other general obesity measurement methods may not accurately assess metabolic status and body fat distribution. Visceral adipose tissue, which is considered a marker of ectopic fat deposition and hormonal environmental disorders, is more metabolically active and potentially reflects the natural metabolic abnormalities of obesity (10). It refers to the adipose tissue accumulated in the peritoneal cavity between the organs and the trunk and is a significant component of total body adipose tissue (11). Increased visceral adipose tissue, also known as central obesity, is an important manifestation of obesity. While previous studies have reported a genetic causal relationship between BMI and knee and hip OA (12), there is limited research on the relationship between visceral adipose tissue and OA, and the precise association between them remains unclear (13).

Mendelian randomization is an analytical method that utilizes genetic variation as an instrumental variable to investigate the causal relationship between exposure and outcomes based on the random distribution of genetic variations during conception (14). This method can largely mitigate the influence of reverse causality and confounding factors in observational studies (15), making it increasingly utilized in clinical studies. The purpose of this study is to explore the relationship between visceral adipose tissue and OA using a two-sample Mendelian randomization research method, aiming to provide insights for managing OA.

## 2 Method and design

### 2.1 Research design

**Figure 1:**
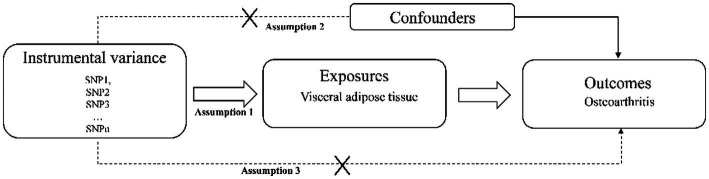
Basic assumptions for Mendelian randomization.

In this study, a two-sample Mendelian randomization analysis was used to select SNPs significantly associated with visceral adipose tissue as instrumental variables to explore the relationship between visceral adipose tissue and all OA, hand OA, hip OA, knee OA, and spine OA. Mendelian randomized research design must meet three assumptions: (1) Instrumental variables are related to exposure factors. (2) Instrumental variables are not related to confounding factors. (3) Instrumental variables can only affect outcomes through exposure factors (16) Figure 1.

### 2.2 Data sources

Visceral adipose tissue-related data were obtained from a recent large-scale summary of GWAS by Karlsson et al. (17) which included 325,153 white British subjects. The study consisted of two cohorts and used estimates by dual-energy X-ray absorptiometry (DXA) to create predictive models. Through screening, we selected the single-nucleotide polymorphism (SNP) that was significantly correlated with VAT (*p* < 5 × 10^−8^) at the whole-gene level. After removing the linkage imbalance and palindrome sequence, the *F*-value of each SNP was calculated, and SNPs with an F-value >10 were selected as tool variables. In addition, we removed SNPs associated with confounding factors (apolipoprotein B, low-density lipoprotein, smoking, and osteoporosis) and outcomes through the online website PhenoScanner.^1^

Data related to OA were obtained from the latest GWAS data (18) of the Osteoarthritis Genetics (GO) Consortium, which included 826,690 samples from 177,517 OA patients. The number of OA cases in the hand, spine, hip, and knee joints was 20,901, 28,372, 36,445, and 62,497, respectively, all of which were European population samples. The basic information included in the data sources is presented in Table 1.

**Table 1:** Data sources related to exposure and outcome.

Exposure or outcome	Participants (Ncase/Control case)	Descent	Source	Pubmed ID
Exposure				
Visceral adipose tissue	325,153	European	https://www.ebi.ac.uk	31501611
Outcome				
All osteoarthritis	17,7,517/649,173	European	https://www.genetics-osteoarthritis.com	34822786
Hand osteoarthritis	20,901/282,881	European	-	34822786
Hip osteoarthritis	36,445/316,943	European	-	34822786
Knee osteoarthritis	62,497/333,557	European	-	34822786
Spine osteoarthritis	28,372/305,578	European	-	34822786

### 2.3 Statistical analysis

The main effect analysis in this study was the inverse variance weighting (IVW) of random effects, which combined the Wald ratio of results for each SNP and conducted a meta-analysis. In addition, MR-Egger regression and a weighted median estimator (WME) were used to supplement the IVW method. Outliers were screened using the MR PRESSO method. If any outliers were found, they were excluded, and MR analysis was performed again. The reliability of the results was tested using sensitivity analysis methods such as Cochran’s Q, MR-Egger intercept analysis, and funnel plot. Cochran’s Q statistics were used to test for heterogeneity. A *p*-value of >0.05 indicates no significant heterogeneity in the analysis. To evaluate the bias for gene pleiotropy using MR-Egger intercept analysis, the closer the regression intercept to 0, the less likely the gene pleiotropy would be. We also generated power values for each MR analysis using an online MR power calculation tool^2^ (19).

All analyses in this study were performed on R 4.2.1 and the MR PERESSO and TwosampleMR packages. After Bonferroni correction, a *p*-value of <0.01 (0.05/5) was considered significant.

## 3 Results

### 3.1 Instrumental variables

After screening, 218 SNPs associated with visceral adipose tissue were identified, explaining approximately 3.38% of the genetic variation. The *F*-values of the included SNPs were > 10, excluding the possibility of weak instrumental variables. The details of the included SNP are shown in the Supplementary material.

### 3.2 MR results

**Figure 2:**
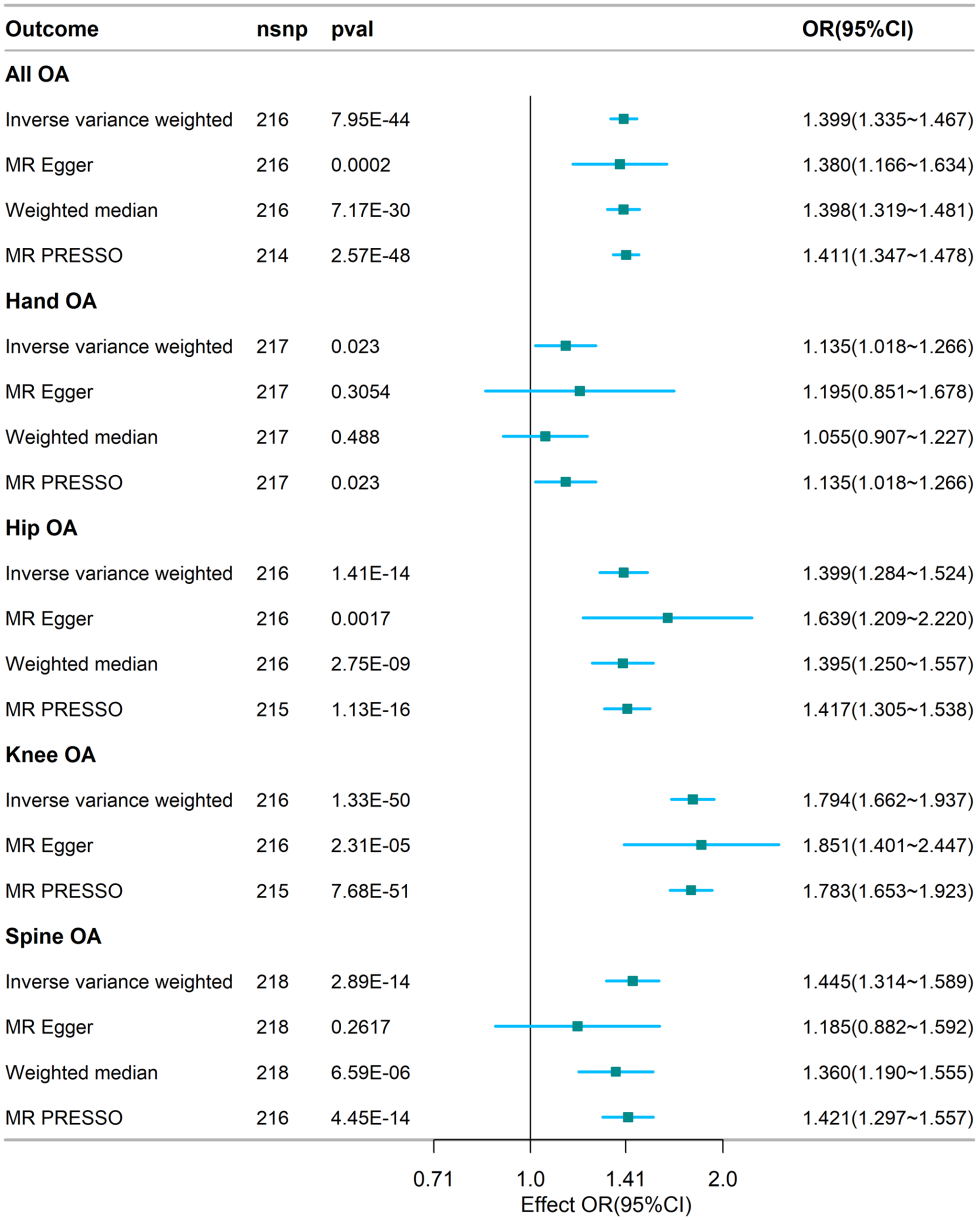
Association between visceral adipose tissue and osteoarthritis based on different methods. OA, osteoarthritis; SNP, single nucleotide polymorphism; IVW, inverse-variance weighted; OR, odds ratio; CI, confidence interval; nsnp, number of snp.

**Figure 3:**
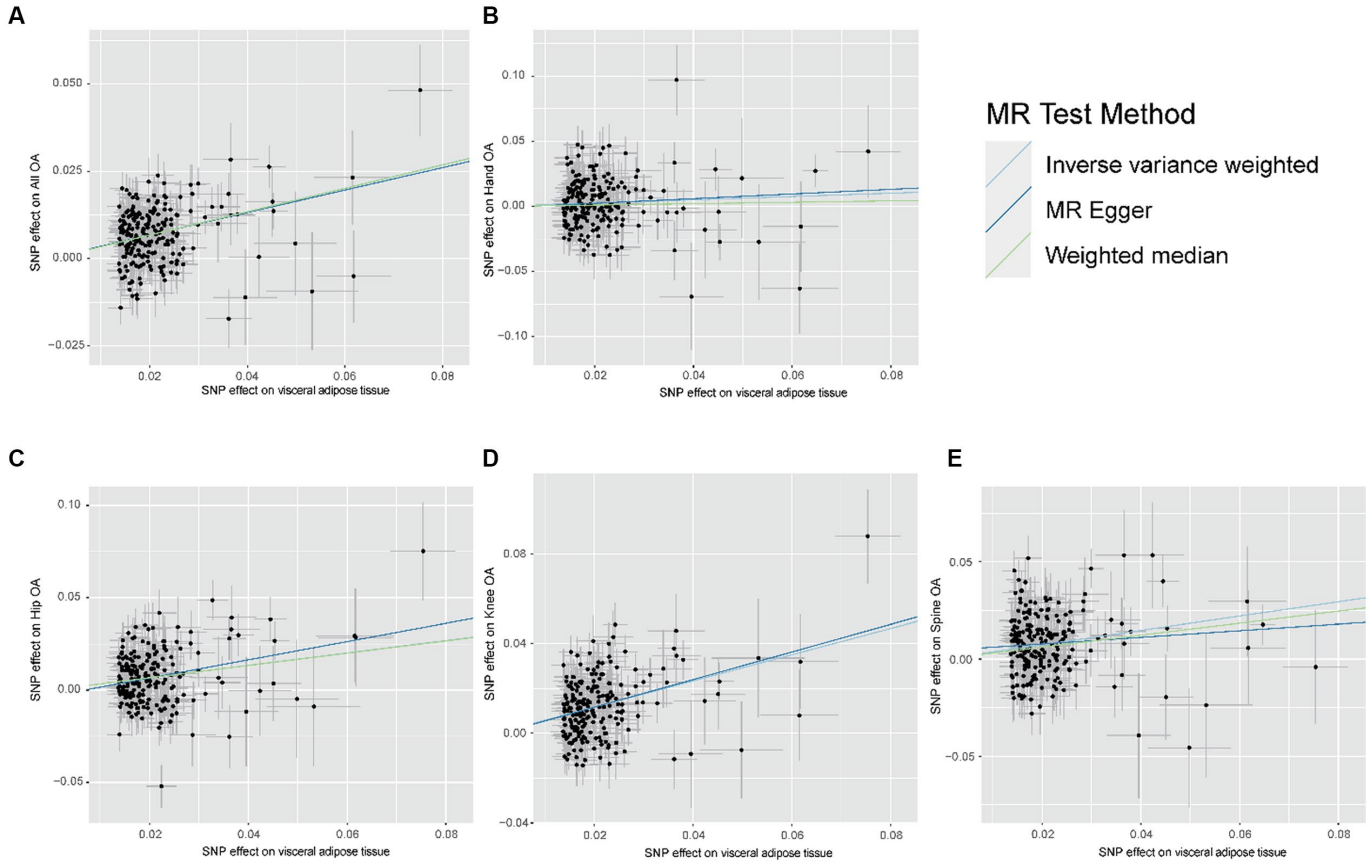
Scatter plot of the relationship between visceral adipose tissue and OA. **(A)** Scatter plot of the relationship between visceral adipose tissue and All OA. **(B)** Scatter plot of the relationship between visceral adipose tissue and Hand OA. **(C)** Scatter plot of the relationship between visceral adipose tissue and Hip OA. **(D)** Scatter plot of the relationship between visceral adipose tissue and Knee OA. **(E)** Scatter plot of the relationship between visceral adipose tissue and Spine OA; OA, osteoarthritis.

The results of the MR analyses are presented in Figure 2. Our results showed that genetically predicted visceral adipose tissue was associated with all OA (OR = 1.399, 95% CI: 1.335–1.467, *p* = 7.95e-44), which was also directionally consistent and significantly validated in the MR Egger, WME, and MR PRESSO methods. In addition, visceral adipose tissue is also associated with hip OA, knee OA, and spinal OA (hip OA: OR = 1.399, 95% CI: 1.284–1.524, *p* = 1.41e-14; knee OA: OR = 1.794, 95% CI: 1.662–1.937, *p* = 1.33e-50; and spine OA: OR = 1.445, 95% CI: 1.314–1.589, *p* = 2.89e-14), indicating that the visceral adipose tissue is closely associated with OA at multiple sites. The scatter plot of the MR analysis of the visceral adipose tissue and OA can be seen in Figure 3.

**Figure 4:**
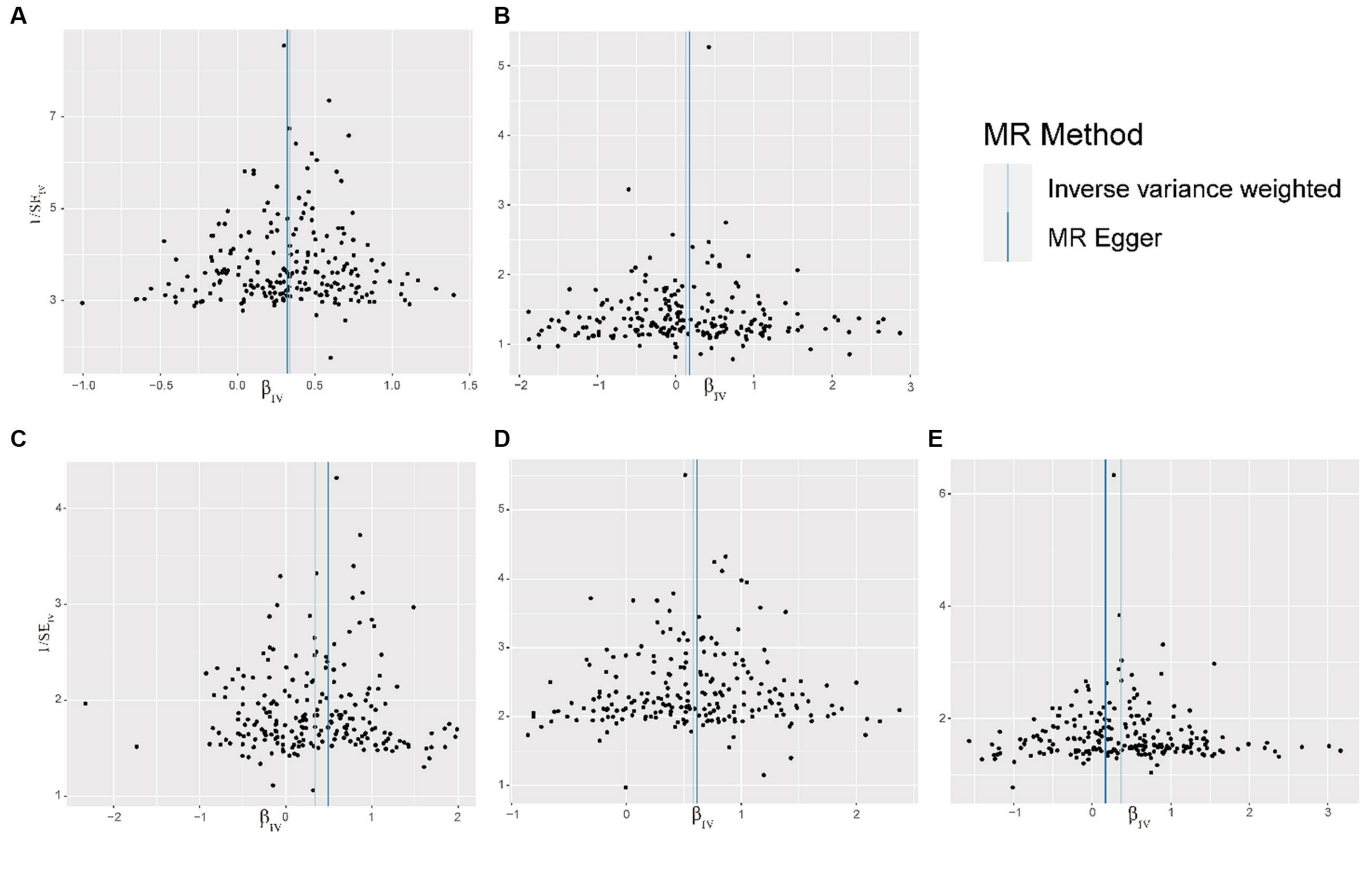
Funnel plot of the relationship between visceral adipose tissue and OA. **(A)** Funnel plot of the relationship between visceral adipose tissue and All OA. **(B)** Funnel plot of the relationship between visceral adipose tissue and Hand OA. **(C)** Funnel plot of the relationship between visceral adipose tissue and Hip OA. **(D)** Funnel plot of the relationship between visceral adipose tissue and Knee OA. **(E)** Funnel plot of the relationship between visceral adipose tissue and Spine OA; OA, osteoarthritis.

The results of sensitivity analyses are presented in Table 2, and in sensitivity analyses, the MR Egger intercept test was used to find potential horizontal pleiotropy. No horizontal pleiotropy was found with each MR analysis. The results of Cochran’s Q-test showed extensive heterogeneity. Because we used random effects IVW as the primary outcome, the heterogeneity was acceptable (20). In addition, no significant bias was observed in the funnel plots of each MR analysis (Figure 4).

**Table 2:** Sensitivity analysis results and power value of the correlation between visceral adipose tissue and osteoarthritis.

	*p* for pleiotropy	Cochrane’s Q	*p* for Cochrane’s Q	Power
All osteoarthritis	0.870	382.2652	9.32E-12	100%
Hand osteoarthritis	0.755	316.6116	9.55E-06	93%
Hip osteoarthritis	0.246	309.1645	2.20E-05	100%
Knee osteoarthritis	0.659	412.9010	9.11E-15	100%
Spine osteoarthritis	0.309	326.1286	1.48E-06	100%

## 4 Discussion

Our study has demonstrated a relationship between visceral adipose tissue and OA. Specifically, for every unit increase in visceral adipose tissue, the risk of developing all OA increases by 40%. Moreover, the risk of hip OA increases by 40%, knee OA increases by 79%, and spine OA increases by 45%. These findings may provide new insights into the connection between OA and obesity.

The association between obesity and OA has been extensively explored in previous studies. Reyes et al.’s cohort study highlighted the association between BMI and OA (21). Similarly, Yuan et al.’s Mendelian randomization analysis indicated that elevated BMI increases the risk of hip OA (22). Another study comprehensively evaluated various measures of obesity, such as waist-to-hip ratio, waist circumference, hip circumference, and body fat content, and their effects on knee and hip OA. This study revealed that different measures of obesity have varying impacts on OA (23). However, these studies did not specifically focus on the influence of visceral adipose tissue on OA. Therefore, our study contributes additional evidence to elucidate the relationship between visceral adipose tissue and OA. To the best of our knowledge, this is the first Mendelian randomized study investigating the connection between visceral adipose tissue and OA.

Prior research has suggested that the accumulation of visceral adipose tissue may be more detrimental than adipose tissue in other body locations (24). In a study by Erdal Belen et al., the thickness of epicardial fat in knee OA patients was found to be greater than that in the control group, and this thickness was associated with the severity of knee OA (25). Furthermore, Eric et al. demonstrated that patients with knee OA exhibited excessive fat accumulation in the central region (26). Although there is no direct evidence linking visceral adipose tissue to OA, Li et al. demonstrated an association between visceral adipose tissue and joint pain (27). Additionally, Visser et al.’s epidemiological study (28) on the Dutch population revealed an association between hand OA and visceral adipose tissue, corroborating our findings.

The link between visceral adipose tissue and OA may be influenced by multiple mechanisms. OA, being a degenerative disease, is believed to be associated with inflammatory processes (29). As the primary fat reservoir in the human body, the visceral adipose tissue is thought to secrete various adipokines, including interleukin-6 (IL-6) and tumor necrosis factor (TNF). These adipokines are believed to play a role in the pathogenesis of OA (30, 31). Interleukin 6 is believed to facilitate cartilage degradation in post-traumatic OA by promoting an increase in MMP-13 and aggrecanase expression. Additionally, its effects are influenced by gender (32). In their study, Xue et al. demonstrated that tumor necrosis factor enables the upregulation of mRNA for a disintegrin and metalloproteinase with thrombospondin motifs 4 (ADAMTS-4), which plays a key role in the pathogenesis of OA by promoting cartilage breakdown in humans (33). Furthermore, leptin, an inflammatory adipose factor, has been shown to affect distal joints. It can enhance collagen degradation and regulate the production of metalloproteinases, thus promoting chondrocyte degradation (34, 35). On the other hand, adiponectin may have a protective effect against OA progression (36), but the accumulation of visceral adipose tissue may inhibit adiponectin transcription, thus enhancing its pro-inflammatory effect (37).

Observational studies are bound to be influenced by confounding factors. However, we have minimized the impact of reverse causality and confounding factors as much as possible by using Mendelian randomization methods. This method provides evidence for the connection between visceral adipose tissue and OA at different anatomical sites. This association has also been verified through sensitivity analysis. Nonetheless, our study has certain limitations. First, due to the constraints of the original GWAS data source, our research primarily encompasses the European population, and we have not explored similar associations in other populations. Second, although we did not identify the presence of level pleiotropy in our study, there was significant heterogeneity among SNPs, and we did not undertake further data filtering to reduce heterogeneity. Third, the original data did not provide age stratification, which prevented us from conducting stratified data analysis to assess the impact of age. Fourth, the proportion of genetic variation explained by visceral adipose tissue remains relatively small. Additionally, our MR analysis may reflect the effect of lifelong exposure to high visceral adipose tissue on OA, yet the risk of OA at a specific time may be influenced differently. Finally, the susceptibility of visceral adipose tissue to OA may be influenced by maternal effects. Intrauterine exposure or maternal behavior, influenced by the maternal genetic background, may contribute to the association between offspring genotype and the risk of OA (38).

## 5 Conclusion

Above all, our study showed that genetically predicted visceral adipose tissue is associated with OA, which also reveals the adverse effects of obesity on human health at the genetic level. Controlling central obesity through intervention is of positive significance for the prevention of OA. However, further large-scale longitudinal studies or randomized controlled trials are needed to further investigate the profound relationship between visceral adipose tissue and the increased risk of OA.

## Data availability statement

The original contributions presented in the study are included in the article/Supplementary material, further inquiries can be directed to the corresponding author.

## Author contributions

YZ: Writing – original draft, Data curation, Methodology, Supervision, Writing – review & editing. YW: Writing – review & editing, Conceptualization, Investigation, Writing – original draft. JX: Data curation, Writing – review & editing. ZW: Methodology, Writing – review & editing. WZ: Supervision, Writing – review & editing. CZ: Funding acquisition, Writing – review & editing.

## Footnotes

^1^ http://www.phenoscanner.medschl.cam.ac.uk

^2^ https://shiny.cnsgenomics.com/mRnd/
